# Interactions of exercise training and high-fat diet on adiponectin forms and muscle receptors in mice

**DOI:** 10.1186/s12986-016-0138-2

**Published:** 2016-11-03

**Authors:** Mélany Pierard, Stéphanie Conotte, Alexandra Tassin, Sébastien Boutry, Pierrick Uzureau, Karim Zouaoui Boudjeltia, Alexandre Legrand

**Affiliations:** 1 Laboratory of Respiratory Physiology, Pathophysiology and Rehabilitation, Research Institute for Health Sciences and Technology, University of Mons, Mons, Belgium; 2 Department of General, Organic and Biomedical Chemistry, Research Institute for Health Sciences and Technology, University of Mons, Mons, Belgium; 3 Center for Microscopy and Molecular Imaging (CMMI), Gosselies, Belgium; 4 Experimental Medicine Laboratory, Free University of Brussels, CHU de Charleroi, Belgium

**Keywords:** Metabolic syndrome, Animal model, Obesity, Multimer, Differential regulation

## Abstract

**Background:**

Metabolic syndrome (MetS) is characterized by systemic disturbances that increase cardiovascular risk. Adiponectin (Ad) exhibits a cardioprotective function because of its anti-inflammatory and anti-atherosclerotic properties. In the bloodstream, this adipocytokine circulates on multimers (Ad_mer_), among which high molecular weight (HMW) are the most active forms. Because alterations of Ad plasmatic levels, Ad_mer_ distribution and receptor (AdipoR) expression have been described in murine models and obese patients, strategies that aim to enhance Ad production or its effect on target tissues are the subject of intense investigations. While exercise training is well known to be beneficial for reducing cardiovascular risk, the contribution of Ad is still controversial. Our aim was to evaluate the effect of exercise training on Ad production, Ad_mer_ distribution and AdipoR muscle expression in a murine model of MetS.

**Methods:**

At 6 weeks of age, mice were submitted to a standard (SF) or high-fat high-sugar (HF) diet for 10 weeks. After 2 weeks, the SF- and HF-fed animals were randomly assigned to a training program (SFT, HFT) or not (SFC, HFC). The trained groups were submitted to sessions of running on a treadmill 5 days a week.

**Results and conclusions:**

The HF mice presented the key problems associated with MetS (increased caloric intake, body weight, glycemia and fat mass), a change in Ad_mer_ distribution in favor of the less-active forms and increased AdipoR2 expression in muscle. In contrast, exercise training reversed some of the adverse effects of a HF diet (increased glucose tolerance, better caloric intake control) without any modifications in Ad production and Ad_mer_ distribution. However, increased AdipoR1 muscle expression was observed in trained mice, but this effect was hampered by HF diet. These data corroborate a recent hypothesis suggesting a functional divergence between AdipoR1 and AdipoR2, with AdipoR1 having the predominant protective action on metabolic function.

**Electronic supplementary material:**

The online version of this article (doi:10.1186/s12986-016-0138-2) contains supplementary material, which is available to authorized users.

## Background

Metabolic syndrome (MetS) is defined as a cluster of disorders (abdominal obesity, insulin resistance, hypertension, dyslipidemia) that lead to an increased risk of type 2 diabetes mellitus, cardiovascular disease and mortality [1]. Its prevalence is cause for increasing concern throughout the world [1].

Adiponectin (Ad) is a 30-kDa protein mainly secreted by adipose tissue. Because this adipocytokine exerts anti-diabetic, anti-inflammatory and anti-atherogenic properties, increasing attention has been paid to the mechanism through which it is modulated in MetS [2]. Hypoadiponectinemia has been associated with both MetS and various pathological states, such as obesity and diabetes. Ad deficiency in mice results in inflammation [3], insulin resistance [4–6] and heart failure [7]. In view of these observations, Ad has been suggested as a promising and easily detectable biological marker for MetS (reviewed in [8]). Ad post-translational modifications result in multimeric forms (Ad_mer_) that are found in the bloodstream and are classified as low (LMW), medium (MMW) and high molecular weight (HMW). HMW forms are commonly considered the most biologically active because they exert more potent AMPK activation and correlate better with insulin sensitivity compared with LMW forms (reviewed in [8]).

Ad is known to regulate lipid and glucose metabolism through its AdipoR1 and AdipoR2 transmembrane receptors [9, 10]. AdipoR1 and AdipoR2 are ubiquitously expressed, with the highest levels occurring in skeletal muscle and the liver, respectively [11]. AdipoR1 has been shown to act predominantly through the AMPK signaling pathway, whereas AdipoR2 initiates PPAR-α intracellular cascades. AdipoR1/AdipoR2 double-KO mice present glucose intolerance and hyperinsulinemia, demonstrating the key roles of these receptors in the physiological regulation of glucose metabolism and insulin sensitivity [10]. However, the respective functions of AdipoR1 and AdipoR2 in vivo are still being investigated [11]. In addition, T-cadherin, a GPI-anchored Ad-binding protein expressed in endothelial cells, macrophages and cardiomyocytes, was identified as an important mediator of Ad’s cardiovascular protective actions [12, 13]. Interestingly, in addition to the variation in Ad plasma levels (Ad_pl_), AdipoRs expression has been reported to be regulated under physiological and pathological conditions. Indeed, an increased AdipoR mRNA level was described in the liver and skeletal muscle after fasting in mice [14]. Moreover, a reduced plasmatic HMW levels and AdipoR1/2 expression in skeletal muscle and adipose tissue were observed in diabetic patients and ob/ob mice, respectively [14, 15]. Molecular mechanisms underlying AdipoR regulation remain poorly known but it was shown that insulin negatively regulates AdipoR mRNA, likely through PI3-kinase/FoxO1 pathway [14]. As well, Park et al. described that endoplasmic reticulum stress inducible factor ATF3 (cyclic AMP-dependent Transcription Factor 3) may also act as a transcriptional repressor of AdipoRs [16]. Post-transcriptional regulations are still currently under investigation and may involve miRNA or alternative splicing [17].

Consequently, strategies that aim to enhance Ad_pl_ and/or activate AdipoR and/or post-receptor signaling pathways are currently the subject of intense study [18]. AdipoR agonists constitute promising pharmacotherapeutic approaches for obesity-related diseases [19]. Among those, AdipoRON [20] is very attractive because of its oral administration, its high absorption and its delivery into relevant target tissues. However, AdipoRON-induced intracellular signaling pathways are not fully characterized, and potential side effects (ventricular hypertrophy, tumor growth, infertility) must be further examined [19].

Although pharmacotherapy plays an important role in the clinical treatment of obesity-associated problems (hypertension, elevated LDL-cholesterol), physical activity is now recognized as a therapeutic strategy for the management of MetS [21, 22]. Exercise training is known to reduce body weight, waist circumference, fat mass (particularly visceral fat), blood pressure and inflammation [21–23]. Exercise training also improves insulin sensitivity [23], lipid profile [24] as well as fasting plasma glucose [22] and enhances β-cell function [21, 24]. However, the contribution of the Ad pathway to these beneficial effects remains unclear. In obese patients, Saunders et al. found an increased Ad_pl_ level after 3 bouts of aerobic treadmill exercise at either low or high intensity over a 1-week period [25]. However, in elderly people with impaired glucose tolerance, Bloem et al. did not observe any Ad_pl_ modification after 7 days of aerobic exercise at 60–70 % of the heart rate reserve, despite improvements in insulin resistance and β-cell function [24]. These controversial effects of exercise training on Ad_pl_ are likely caused by variations in the training program (type, duration, intensity) and different pathological contexts. Another point to be considered in those discrepancies is the lack of data regarding Ad_mer_ distribution and AdipoR expression. Indeed, a recent study of Ad-KO mice suggested that the absence of Ad does not impair the capacity of an 8-week endurance training program to increase glucose and insulin tolerance [26]. Although AdipoR expression has not been investigated, compensatory mechanisms involving AdipoR1 were hypothesized to explain the maintenance of insulin sensitivity [26].

In brief, when exercise training is beneficial in MetS, the contribution of Ad to this effect is still controversial. Compensatory mechanisms involving the AdipoR receptor have been suggested, but they seem to depend on training intensity and duration. Therefore, to clarify the consequences of longer-term aerobic exercise on Ad actions in the context of MetS, we investigated the effect of an 8-week aerobic treadmill training program on Ad production via the assessment of plasmatic Ad_mer_ distribution and AdipoR1/AdipoR2 muscle expression in high-fat diet (HF)-fed mice.

## Methods

All procedures met the Belgian national standard requirements regarding animal care and were conducted in accordance with the Ethics and Welfare Committee of the University of Mons.

### Animals and diet

The experiments were performed on male C57BL6J mice bred in our animal facility (accreditation number LA1500022). The mice were housed in cages with ad libitum access to water and food and were maintained at 35–40 % relative humidity and a temperature of 20–23 °C in a 12:12 h light–dark cycle. At the age of 6 weeks, the animals were randomized to a standard (SF) or a high-fat high-sugar (HF) diet. In addition to a standard vitamin and mineral mix, the SF group received pellets consisting of 70 % energy from complex carbohydrates (corn starch), 20 % protein, and 10 % fat (D12450K-Research Diet, Inc., New Brunswick, NJ, USA), whereas the HF diet was composed of 20 % refined carbohydrates (7 % sucrose and 13 % maltodextrin), 20 % protein, and 60 % saturated and mono-unsaturated fat, primarily from lard (D12492-Research Diet, Inc., New Brunswick, NJ, USA). Food intake and body weight were measured once a week during a 10-week exposure period. The day following the end of the protocol, mice were sacrificed, blood and tissues were collected for RT-qPCR, ELISA and western blot analysis.

### Exercise training protocol

After two weeks (at the beginning of week 3), the SF and HF animals were randomly assigned to exercise-trained (respectively SFT and HFT) or untrained (SFC and HFC) groups. Control mice were not exposed to exercise session and stayed in their cages during the protocol. The mice were exercised on a treadmill (Treadmill Control LE8700, Panlab apparatus®, Barcelona, Spain), 5 days a week for 8 weeks. They were acclimated to the treadmill at 3 m/min for 5 min and 9 m/min for 10 min during weeks 3 and 4. At the beginning of week 5, an incremental test was performed with a gradual speed increase of 1.2 m/min every 2 min. For each mouse, exercise was considered maximal and the test was interrupted when the animal was unable to continue running at the belt speed despite receiving four electric stimulations in one minute. From weeks 5 to 10, the belt speed during training was set at 70 % of the maximal running velocity, and the exercise duration was increased by 10 min per week until a maximum of 60 min was reached. Both trained groups were running at the same velocity. Mean ± SEM of the 70 % of the maximal running velocity of trained groups are detailed in the supporting information (Additional file 1: Table S1).

### Glucose tolerance test

After an overnight fast and 18 h after the last exercise session, a glucose tolerance test (GTT) was performed before the protocol, after week 2 of the protocol and at the end of the protocol. A dose of 2 g/kg body weight of D-glucose (Roth, Karlsruhe, Germany) was administered intraperitoneally. Blood samples were then obtained from the caudal vein, and the blood glucose level was measured 0, 30, 60, and 120 min after glucose injection using a One Touch® Vita® glucometer (Zug, Switzerland).

### MRI

Fat mass and lean body mass were measured using magnetic resonance imaging (MRI) analysis (PharmaScan 7 T, Bruker®, Billerica, MA, USA) at the same time-points as the GTT. The animal was anesthetized by 1.0–2.5 % isoflurane and restrained within a mouse-sized tube. Acquisition was synchronized with the respiratory cycle to minimize physiological artefacts. The region of interest was located between the upper pole of the kidney and the feet. The imaging parameters for the 3D Fisp sequence are detailed in the supporting information (Additional file 1: Table S2). All the 3D images were segmented manually (muscle) or automatically (fat) by the same operator using Slicer 3D. Threshold levels were defined to discriminate the tissue of interest (muscle: 6000–13,000, fat <12,500) and the corresponding voxels were quantified.

### RNA extraction – reverse transcription and real-time PCR

The total RNA from frozen visceral adipose tissue was extracted using the miRNeasy Micro Kit (Qiagen®, Hilden, Germany) according to the manufacturer’s instructions. The same amount of RNA was reverse transcribed into cDNA with SuperScript® III First-Strand Synthesis SuperMix (Invitrogen™, Carlsbad, CA, USA). The qPCR was performed with Lightcycler 480 Real-Time PCR II (F. Hoffmann Roche®, Ltd., Basel, Switzerland). The cycling conditions were as follows: 30 s at 92 °C, 40 cycles of 30 s at 64 °C, and 15 s at 72 °C. All samples were run in duplicate. The primers used for Ad and GAPDH are detailed in the supporting information (Additional file 1: Table S3). The target gene cycle threshold (Ct) was normalized to the expression of the housekeeping gene GAPDH, and gene expression was calculated using the δδCt method.

### ELISA

The Ad and leptin concentrations were measured according to the manufacturer’s instructions (Ad: MRP300, leptin: MOB00, R&D Systems, Minneapolis, MN, USA).

### Western blot

The relative amounts of LMW, MMW and HMW Ad_mer_ were determined using a non-denaturing PAGE-SDS followed by a Western blot. To this aim, 5 μl of plasma diluted to contain 5 μg/ml of Ad was loaded onto 6 % polyacrylamide gel in the presence of SDS. The AdipoR expression level was determined on frozen muscle tissue (*gastrocnemius*) homogenized in a lysis buffer (Cell lytic MT Mammalian Tissue Lysis/Reagent, Sigma-Aldrich, St. Louis, MO, USA) containing a protease inhibitor cocktail (Sigma-Aldrich, St. Louis, MO, USA). Fifty micrograms of total protein extract were then separated using a 12 % denaturant PAGE-SDS. A peptide competition assay was performed to confirm the specificity of each AdipoR antibody. To this aim, the antibody was pre-incubated with or without the peptide prior to the Western blot experiments. Moreover, cross-reactivity with the appropriate AdipoR was also controlled for each antibody. For the Western blot, proteins were transferred to a nitrocellulose membrane (Millipore, Darmstadt, Germany). After blocking with 5 % fat-free dry milk-TBS, the membranes were incubated with rabbit polyclonal primary antibody directed against Ad (Ab85827, 1:1000, Abcam, Cambridge, UK), against AdipoR1 (1:1000) or AdipoR2 (1:750) (AdipoR12-A, AdipoR22-A; Alpha Diagnostic, San Antonio, TX, USA). For standardization, the membranes were stripped, and immunostaining was performed with a mouse polyclonal antibody against GAPDH (Am4300, 1:5000, Invitrogen™, Carlsbad, CA, USA). The membranes were then incubated with a horseradish peroxidase-labeled secondary antibody (1:5000, Sigma-Aldrich, St. Louis, MO, USA). The ECL™ Western Blotting Detection kit (GE Healthcare, Little Chalfont, UK) was used for the revelation step. The immunoreactive bands were then submitted to a densitometric analysis using the Image J software.

### Statistical analysis

For the sake of comparison, food intake values were averaged for the first 2 weeks (week 2) and the last 8 weeks (week 10). Using a least-square linear regression, we evaluated the change in body weight over time from week 0 to week 10 and calculated the slope coefficient for each animal. Lean and fat mass values are presented as the ratios of week 2/week 0 and week 10/week 2 (considered the week 2 and week 10 values, respectively). The statistical analyses of lean body mass, fat mass and body weight were determined using a Mann-Whitney rank sum test (week 2). Food intake and glucose tolerance were assessed using Student’s t-test (week 2) and one-way ANOVA followed by Holm-Sidăk test (week 10). The ANOVA on ranks was used to determine the statistical significance of the slope body weight, body weight at week 10, lean and fat mass (week 10), Ad mRNA, Ad_pl_ level and Ad_mer_ distribution. Finally, AdipoR1 and AdipoR2 muscle expression were compared using two-way ANOVA followed by Holm-Sidăk test. Differences were considered statistically significant at a *P value* < 0.05. All data were represented as mean ± sem or boxplot (5 and 95th percentile) for parametric or non-parametric statistical tests, respectively.

## Results

### Exercise training limits food intake and prevents body weight gain and fat mass accumulation in HF-fed mice

**Fig. 1:**
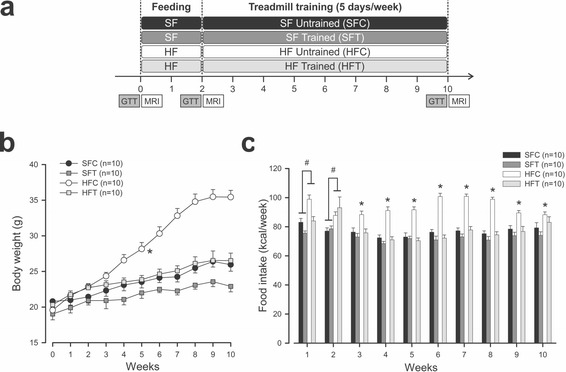
Effect of diet and exercise training on body weight and food intake. **a** Timeline. **b** Body weight evolution. Mean ± sem. * *p* < 0.001 slope comparison, HFC vs all groups, ANOVA on ranks. **c** Food intake evolution. Mean ± sem. # *p* < 0.001, HF vs SF, t-test; * *p* < 0.001, HFC vs all groups, One way ANOVA. GTT: glucose tolerance test, MRI: magnetic resonance imaging, HF: high-fat high-sugar diet, SF: standard diet

To evaluate the effect of exercise training, standard (SF) and high-fat high-sugar (HF) fed animals were submitted to a training program (SFT and HFT) and compared to control animals (SFC and HFC). The experimental design is presented in Fig. 1a. The evolution of body weight and food intake are illustrated in Fig. 1b and c, respectively. Compared with controls, the HF mice had significantly increased food intake during weeks 1 and 2 (Fig. 1c, *p* < 0.001). During the training period, this difference was only maintained in the non-exercising HFC animals (Fig. 1c, *p* < 0.001). This was associated with a greater increase in body weight at the end of week 2 in the HFC and HFT groups. During the training period, the slope of the relationship of body weight over time was significantly higher for the HFC group compared with the three other groups (Fig. 1b, *p* < 0.001).

**Fig. 2:**
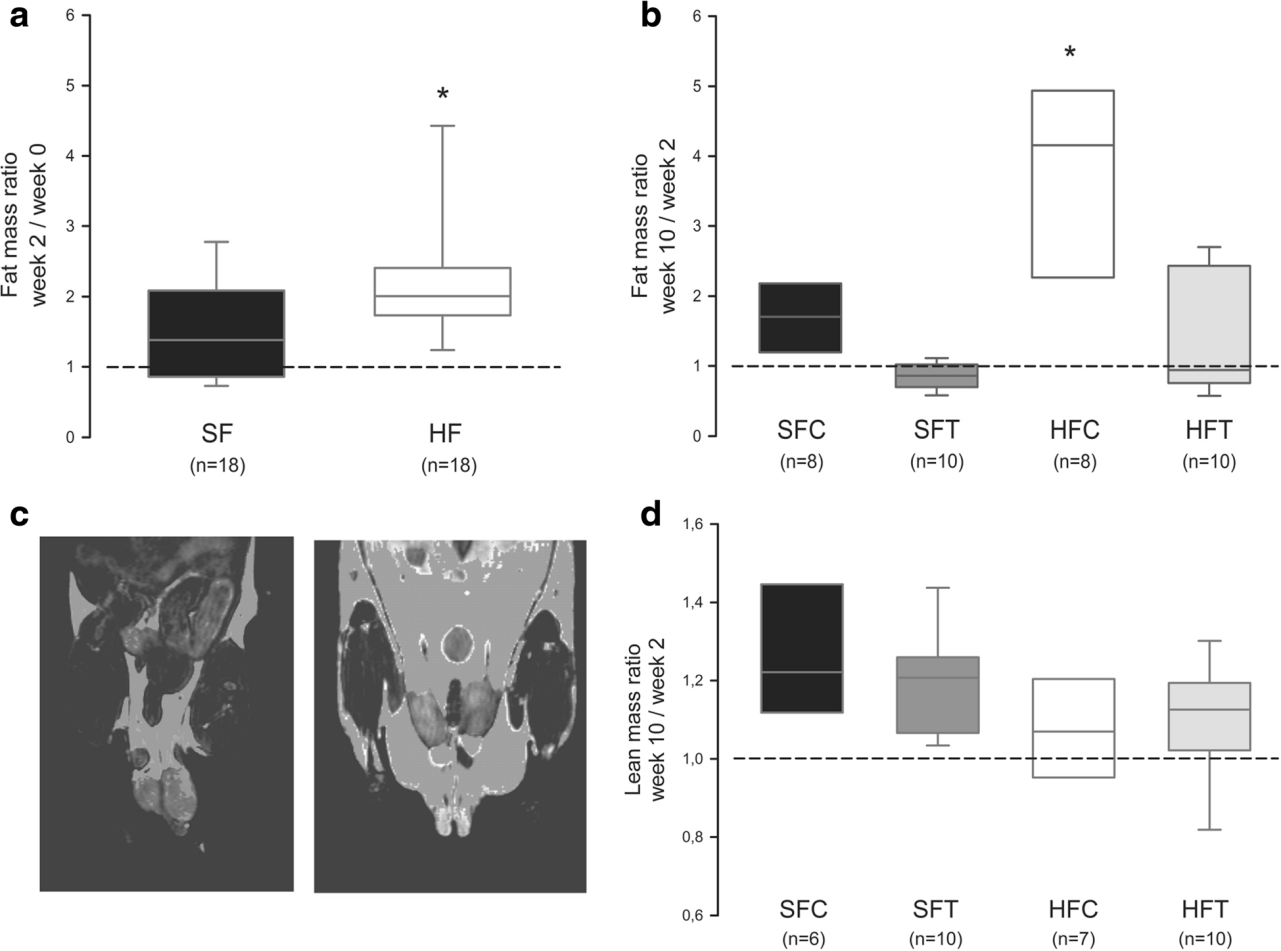
Effect of diet and exercise training on fat and lean mass. **a** Fat mass evolution during the untrained period. Fat mass was measured using MRI, and the ratios are represented as boxplots (5 and 95th percentile). * *p* < 0.05, HF vs SF, Mann-Whitney rank sum test. **b** Fat mass evolution between weeks 2 and 10. Ratios are represented as boxplots (5 and 95th percentile). * *p* < 0.001, HFC vs SFT and HFT, ANOVA on ranks. **c** Representative MR Images obtained for SF- (*left*) and HF-fed (*right*) mice. Positive signal for adipose tissue. **d** Lean mass evolution between weeks 2 and 10. Ratios are represented as boxplots (5 and 95th percentile). ANOVA on ranks: NS

To determine whether the observed body weight variations reflected modifications in fat or lean body mass, MRI analyses were performed. When the fat mass at week 2 was expressed as a percentage of the value at week 0, a higher increase was observed in the HF groups compared with the SF groups (Fig. 2a, *p* < 0.05). When the same ratio was calculated for the end to the beginning of the training period, the change in fat body mass was significantly reduced in the HFT group compared with HFC group, and the HFT group was not different from the SF groups (Fig. 2b, *p* < 0.001). The lean body mass was not modified by HF diet or training (Fig. 2d). As expected, our training conditions were able to reverse the adverse effects of an HF diet on body weight through a limitation of fat mass accumulation.

### Exercise training improves the glucose tolerance of HF-fed mice

**Fig. 3:**
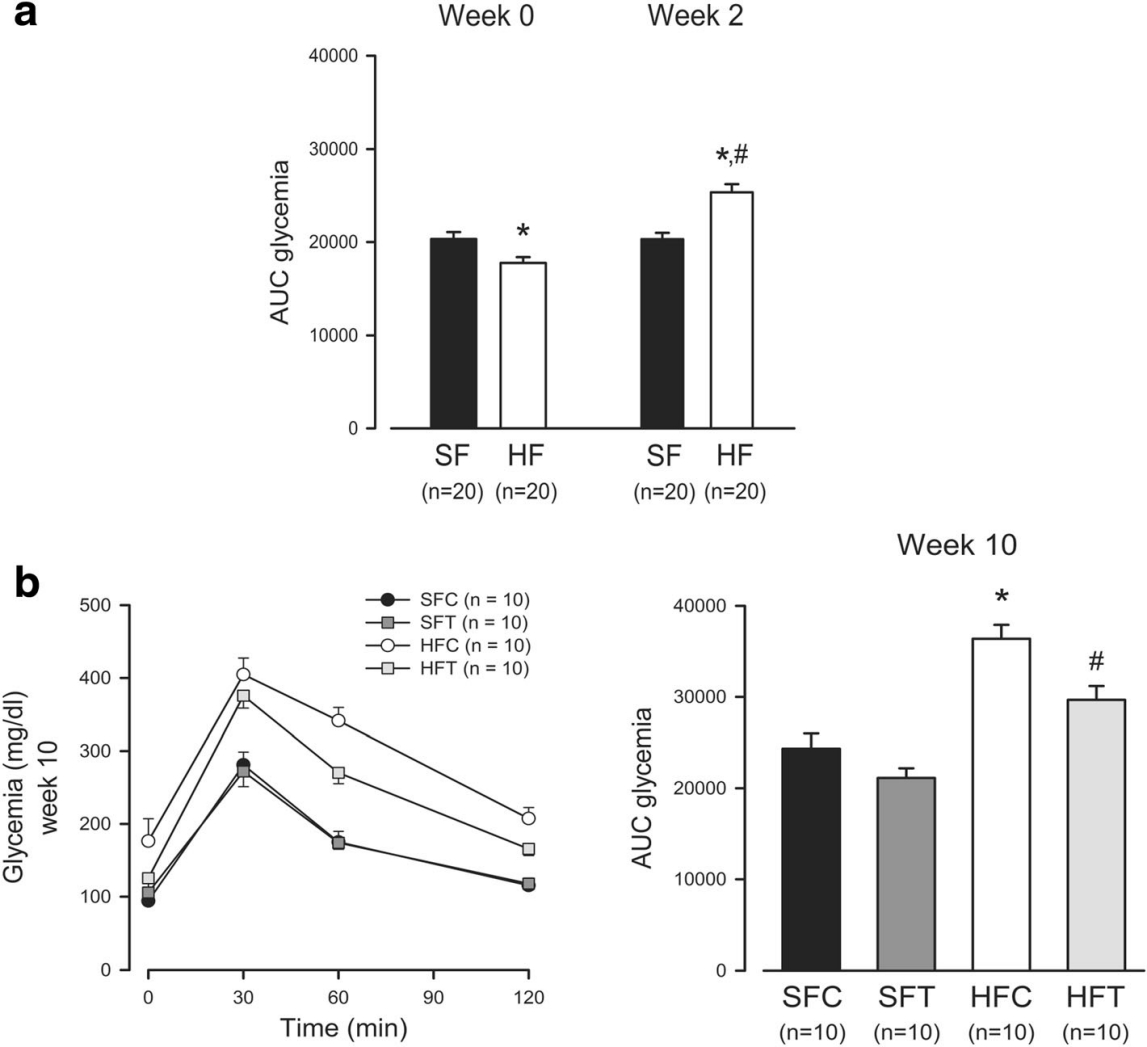
Effect of diet and exercise training on glucose tolerance. **a** Change in GTT after 2 weeks of an HF diet. Fasted mice were submitted to an intraperitoneal injection of glucose (2 g/ kg b.w.). Glycemia was measured before (0) and 30, 60 and 120 min after injection. Histograms represent the area under the curve (AUC) of glycemia from 0 to 120 min. Mean ± sem. * *p* < 0.05, HF vs SF, t-test; # *p* < 0.001, HF week 2 vs HF week 0, paired t-test. **b** GTT at week 10. The left panel represents glycemia at the different time-points, and the right panel represents the AUC. Mean ± sem. * *p* ≤ 0.002, HFC vs all groups; # *p* < 0.05, HFT vs all groups, one-way ANOVA

After 2 weeks of the HF diet (Fig. 3a), the AUC of glycemia during the GTT was increased (HF group week 2 vs week 0, *p* < 0.001; HF vs SF group week 2, *p* < 0.05) but was not altered under the standard diet (SF group week 2 vs week 0, NS). At week 10, the AUC of the HFC group was higher than that of the three other groups (Fig. 3b, *p* < 0.002). When exercise training was associated with an HF diet, the increase in the AUC was limited but was not restored to the same level as that of the SF diet groups (*p* < 0.05).

### HF diet induced an Ad_mer_ redistribution that was not reverted by exercise training

**Fig. 4:**
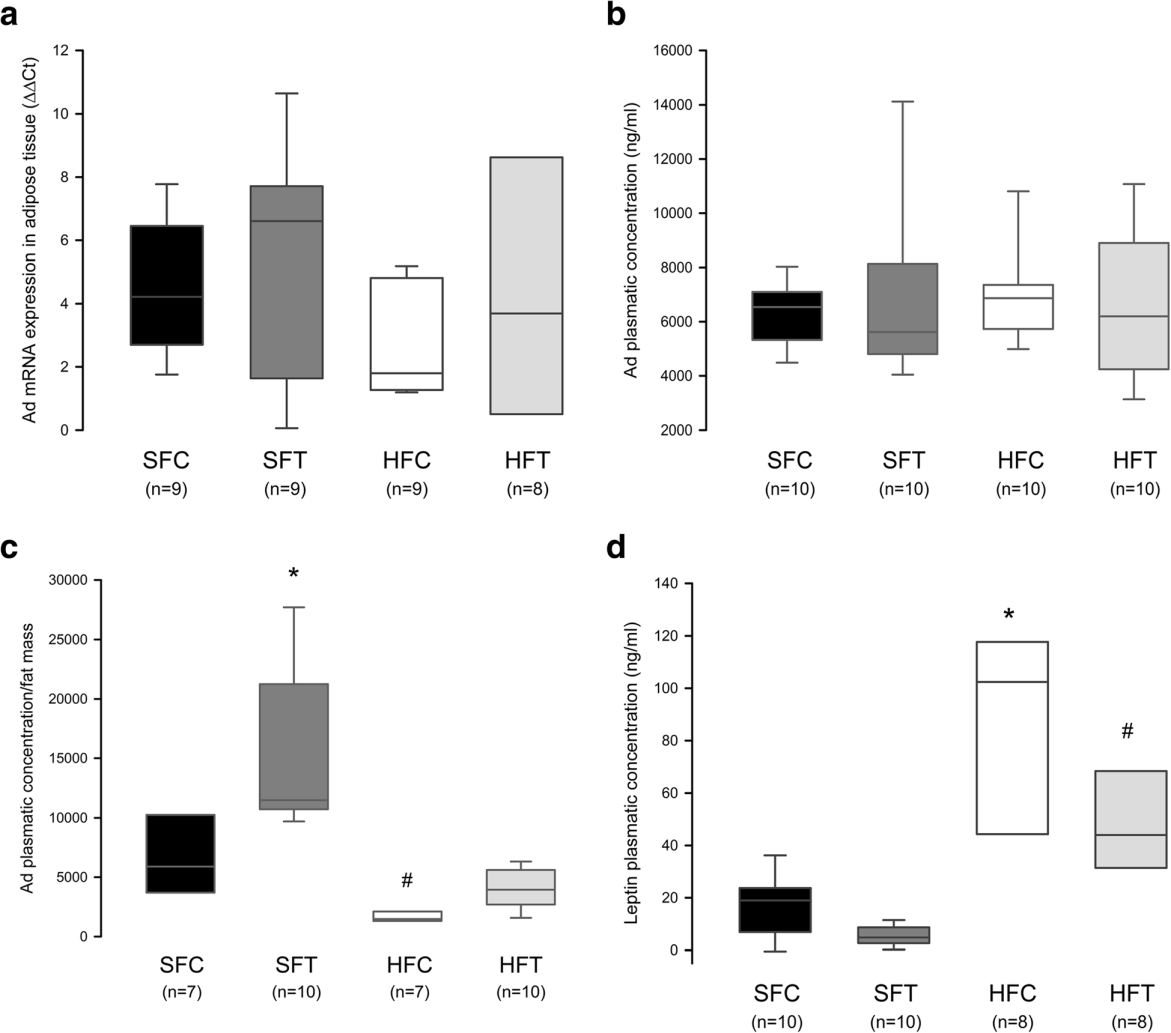
Ad and leptin analysis at week 10. **a** Ad expression in adipose tissue. The Ad mRNA level in abdominal fat was determined by RTqPCR (Syber Green). Data are represented as boxplots (5 and 95th percentile). ANOVA on ranks: NS. **b** and **c** Ad plasmatic level. The total Ad_pl_ concentration was measured using indirect ELISA. Data are represented as boxplots (5 and 95th percentile). Panel b) ANOVA on ranks: NS. Panel c) Ad_pl_ level normalized for fat mass. * *p* < 0.001, SFT vs HFT and HFC; #: *p* < 0.001, HFC vs SFC; ANOVA on ranks. **d** Leptin plasmatic level. The leptin concentration was measured using indirect ELISA. Data are represented as boxplots (5 and 95th percentile). * *p* < 0.001, HFC vs SFC and SFT; #: *p* < 0.001, HFT vs SFT; ANOVA on ranks

To clarify whether the beneficial effects of the aerobic exercise program were associated with modulations of Ad expression or secretion, we first evaluated the Ad mRNA expression in visceral adipose tissue and total Ad_pl_ levels. We did not observe any modification with an HF diet or training (Fig. 4a-b), except when the Ad_pl_ data were expressed in proportion to fat mass (Fig. 4c, *p* < 0.001). Ad production and secretion adjusted in this manner were reduced with the HF diet and improved with training, but the total level of circulating Ad was not modified. In parallel, we found an increased leptin concentration in HF-fed mice compared with SF groups (Fig. 4d, *p* < 0.001). Upon training, leptin level in HF-fed mice was still higher compared with SFT group but not statistically different from control mice.

**Fig. 5:**
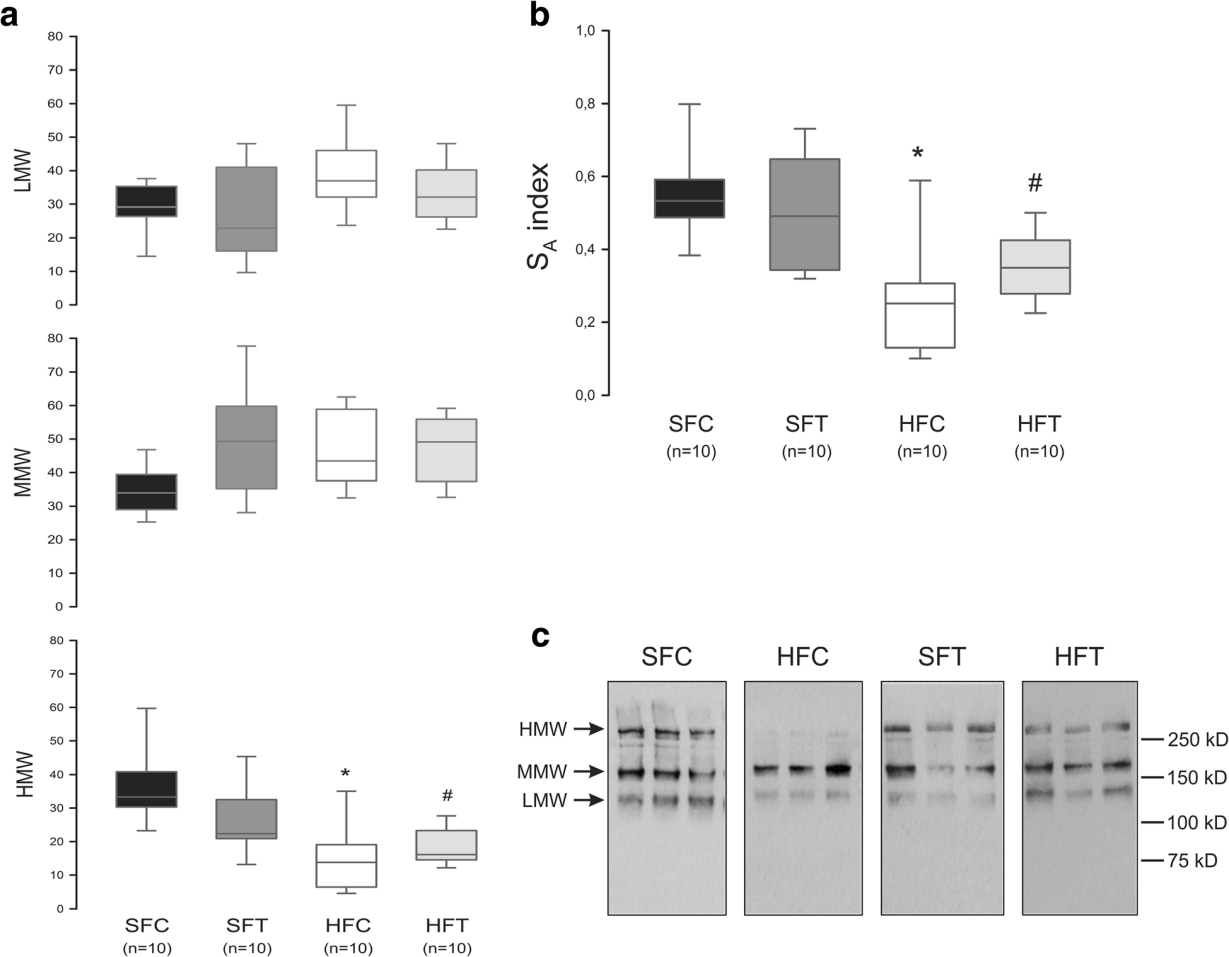
Ad_mer_ distribution analysis at week 10. The proportions of high (HMW), medium (MMW) and low (LMW) molecular weight Ad_mer_ were determined using non-denaturant PAGE-SDS followed by a Western blot. **a** Ad_mer_/total Ad ratio obtained after densitometric analysis. Data are represented as boxplots (5 and 95th percentile).*, # *p* < 0.001 vs SFC, Mann-Whitney rank sum test. **b** S_A_ index was calculated as the ratio HMW/(HMW + LMW). Data are represented as boxplots (5 and 95th percentile).* *p* < 0.001 HFC vs SFC and SFT; # *p* < 0.001, HFT vs SFC, Mann-Whitney rank sum test. **c** Representative blots

Because Ad circulates in different multimeric forms, we quantified the plasmatic proportion of LMW, MMW and HMW Ad_mer_. We observed that the proportion of HMW forms decreased in the HF-fed mice compared with control animals (Fig. 5a, *p* < 0.05). This reduction seemed to be in favor of the LMW forms as shown in Additional file 1: Figure S1a. By contrast, the distribution was not different in the trained and untrained groups (Fig. 5a and Additional file 1: Figure S1b). We also calculated the S_A_ index, defined as the ratio HMW/(HMW + LMW), and reported as a more relevant indicator of insulin sensitivity [27]. We found a reduction of S_A_ index in HF-fed mice compared with control mice (Fig. 5b), in accordance with a reduction of HMW in favor of the LMW forms. However, the effect of training was not sufficient to counteract this redistribution induced by an HF diet.

### HF diet and exercise training are responsible for distinct modulation of the AdipoR muscle expression pattern

**Fig. 6:**
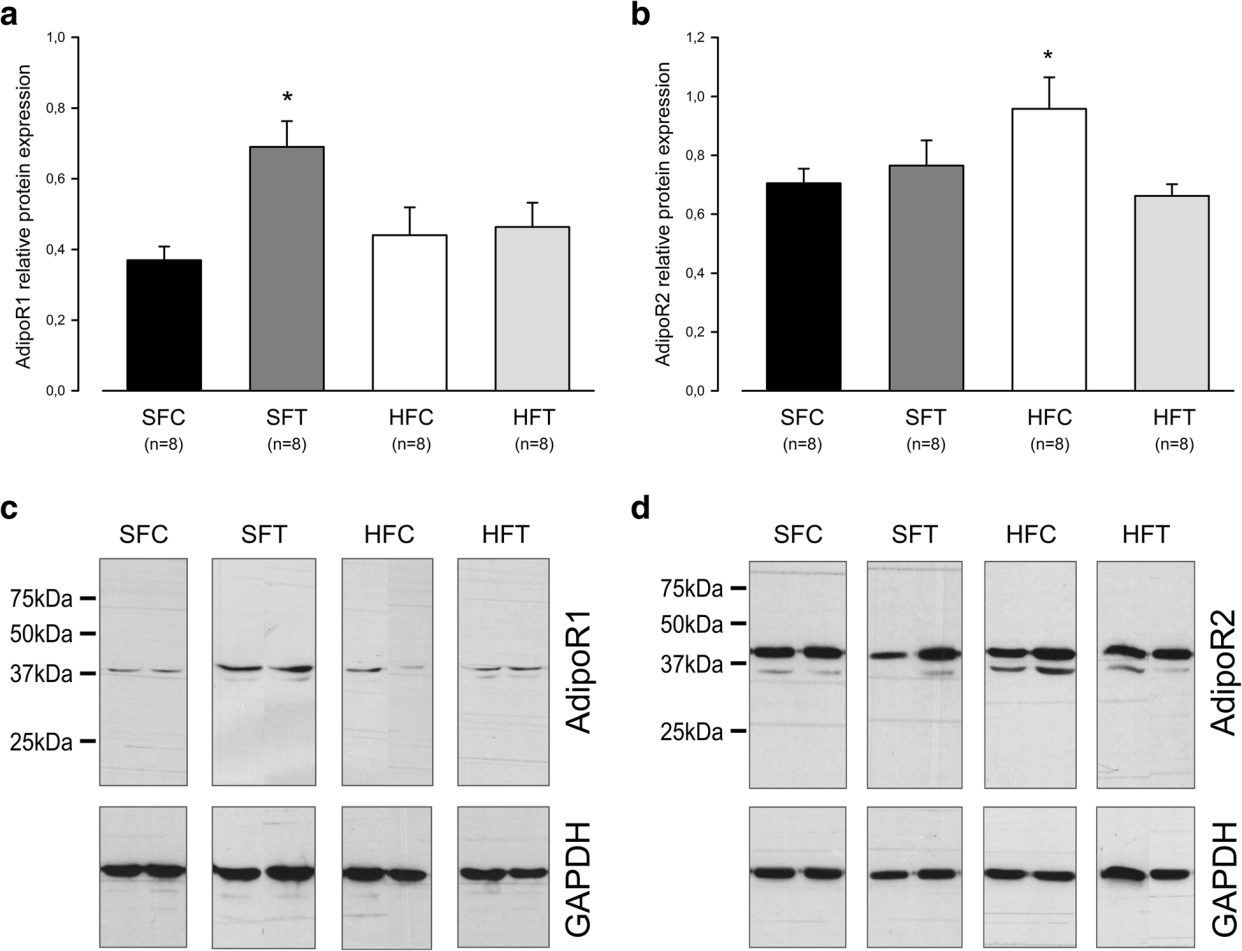
AdipoR muscle expression analysis at week 10. **a** and **c** The relative expression of AdipoR1 was determined using a PAGE-SDS followed by a Western blot using specific antibodies. GAPDH was used as the loading control. The upper panel represents the results obtained after densitometric analysis. The lower panel shows representative blots. Mean ± sem. * *p* < 0.05, SFT vs SFC and HFT, two-way ANOVA followed by Holm-Sidăk test. **b** and **d** The relative expression of AdipoR2 was determined as described in (**a**). Mean ± sem. * *p* < 0.05, HFC vs HFT and SFC, two-way ANOVA followed by Holm-Sidăk test

Exercise training induced a significant increase in the AdipoR1 muscle level in mice submitted to a standard diet (Fig. 6a, *p* < 0.05). However, no difference was observed between the trained or untrained HF - fed mice, suggesting that the induction of an increase in AdipoR1 protein via exercise training is impaired by an HF diet. In contrast, we observed a significantly increased AdipoR2 expression level in the sedentary HF-fed mice compared with the SF-fed animals (Fig. 6b, *p* < 0.05). This effect of an HF diet on AdipoR2 muscle expression is no longer observed when exercise training is performed simultaneously.

## Discussion

### Exercise training can counteract the adverse effects of an HF diet in mice

MetS is a growing public health burden throughout the world that can be treated by changing lifestyle behaviors. Among these, exercise training is well known as a major therapeutic strategy against MetS. In this study, mice exposed to 10 weeks of an HF diet recapitulated the key problems of MetS [28]: an increased caloric intake, reflecting reduced satiety control, as well as increased body weight and fat mass associated with impaired glucose tolerance. Paradoxically, these disturbances were associated to an increased plasmatic leptin level, a hormone reducing food intake and increasing energy expenditure, insulin sensitivity and fat deposition [29]. These results could be explained by a leptin resistance, preventing its central and peripheral functions [30]. In mice that consumed the same HF diet in association with aerobic exercise training, a modification of nutrition-related behavior was observed that resulted in better food-intake and body-weight control. While the increased energy expenditure associated with exercise could participate to its beneficial effects, an improvement of appetite control during chronic exercise is also well described in the literature [31, 32]. Although mechanisms remain to be elucidated, modulations of fat-free mass and fat mass as well as change in hormones such as leptin become recognized as the most important components involved in energy intake regulation during physical activity [33]. In our study, we observed an attenuation of hyperleptinemia upon training, in keeping with an improvement of leptin regulation induced by a higher energy expenditure [29, 34]. Associated to the better food intake control in trained HF mice, our data are in accordance with other studies suggesting that exercise training reduces leptin resistance [35–37].

As expected, we also found in trained HF animals that fat mass was maintained at the level of the controls, and glucose intolerance was partly but significantly improved. These observations corroborated the results of Gomes et al., showing that exercise ameliorated insulin hypersecretion from pancreatic islets of HF diet rats [38]. An increased GLUT4 expression was also observed in trained subjects with MetS [39, 40] and could participate to the decreased glucose intolerance. These studies coupled with our results confirmed that physical activity provides numerous health-related benefits that prevent the development of MetS, whether directly through its effect on skeletal muscle and energy expenditure or indirectly by changing nutrition behavior. In this context, the contribution of Ad remains to be shown.

### HF feeding alters Ad production and modifies Ad_mer_ distribution

Ad_pl_ is known to be inversely correlated with body weight and fat mass (especially visceral fat) and positively correlated with HDL concentration [41], as shown in obese patients compared with lean subjects [42–44] and in HF-fed mice compared with animals consuming a standard diet [45]. In our study, the Ad production per unit of fat mass was reduced with the consumption of an HF diet, suggesting adipose tissue dysfunction. However, the HF diet did not modify the total level of circulating Ad, likely because of the concomitant increase of fat mass and therefore of the number of Ad-producing cells. These results were corroborated by Barnea et al. [46] and Ribot et al. [47], who did not find any Ad_pl_ variation after exposure to an HF diet. In these studies, by adjusting Ad levels to the weight of the white adipose tissue mass, the authors revealed a significant reduction of serum Ad in HF-fed mice. This reduction was associated to an increased circulating leptin level in HF-fed mice. While Ad and leptin were described as two independent factors which are dysregulated in MetS [48], recent studies found that leptin could induce Ad expression in differentiated human white preadipocytes [49], in ob/ob [50, 51] and in LDLR^-/-^ mice [51]. Singh et al. also suggested that leptin resistance may contribute to the reduced Ad expression in obese patients [49]. However, further in vivo and clinical investigations are necessary to confirm any causal relationship between leptin level and Ad expression. Despite the absence of variation in the absolute amount of Ad in the bloodstream, we observed a modification of Ad_mer_ distribution that was characterized by a decreased level of HMW forms in favor of LMW multimers. This decrease was also observed by Anderson et al. [52] and was suggested by Nakashima et al. to predict the existence of MetS [53]. In accordance with this reduction of HMW forms, we observed a reduction of S_A_ index in HF-fed mice compared with control mice. These results were corroborated by Pajvani et al. [27] which observed a decreased S_A_ index in *db/db* mice and in type II diabetic patients compared with control mice and insulin-sensitive individuals, respectively, despite similar total Ad levels. These data reinforce the importance of investigating Ad_mer_ distribution in the diagnosis of MetS, even in the absence of a change in the total Ad_pl_ level [54].

### HF feeding increases muscle expression levels of AdipoR2 but not AdipoR1

Regarding AdipoR expression with HF feeding, Blüher et al. [55] suggested that AdipoR2 upregulation could be a compensatory mechanism in response to reduced Ad_pl_. This hypothesis was reinforced by Bauche et al. [56], who suggested the existence of a regulatory feedback loop by which Ad downregulates its own production and its AdipoR2-receptor expression in adipose tissue. Interestingly, we found an increased AdipoR2 expression in HF-fed mice in the absence of any Ad_pl_ modification. Therefore, it seems reasonable to hypothesize that this modified AdipoR level could be caused by compensatory mechanisms in response to a reduced level of HMW forms.

In contrast to AdipoR2, the AdipoR1 muscle expression level was not modified with the HF diet. This differential effect of HF feeding draws attention to the distinct function of these receptors. Parker-Duffen et al. [11] recently observed that AdipoR2-deficient mice submitted to hindlimb ischemic surgery exhibited severely attenuated revascularization, whereas AdipoR1-deficient mice exposed to an HF diet developed metabolic perturbations characterized by a greater body weight and fat mass, hepatic steatosis, impaired glucose tolerance. These results were corroborated by previous studies observing that AdipoR1-KO mice exhibited increased glucose intolerance after HF feeding [57], whereas AdipoR2-KO mice were resistant to these effects [57, 58, 10]. In concordance with these studies, the increased AdipoR2 muscle expression observed here was not able to counteract the adverse effects of an HF diet on glucose homeostasis and fat mass in the absence of a change in AdipoR1 expression. Different mechanisms have been suggested to explain the different functionalities of AdipoR [2 11]: (i) AdipoR1 and AdipoR2 exert their effects through different signaling pathways: AMPK and PPAR-α, respectively; (ii) potential Ad-binding proteins, such as the adaptor proteins calreticulin and T-cadherin, could modulate AdipoR functions; (iii) The AdipoR1 and AdipoR2 expression pattern and function have been reported to be tissue-specific.

### An 8-week exercise training program has limited effects on Ad_pl_ and Ad_mer_ distribution with an HF diet

In our study, the altered Ad_pl_-to-fat mass ratio suggested that Ad secretion by adipose tissue was improved by training. The total level of circulating Ad, however, was not modulated. Such results are often discussed in terms of body weight variation during the training period. Indeed, a threshold value of 10 % body weight reduction was suggested to be required before increased Ad_pl_ would be observed [59–61]. This was contradicted by Saunders et al. [25], who found that in humans, physical activity was able to increase Ad_pl_ without any change in body weight. Our study design associated a training program with HF feeding and the trained HF mice did not exhibit any weight loss throughout the protocol. Ad level was thus not influenced by a declining body weight during the training program. Trained HF mice maintained their body weight at the level of control animals, most probably due to the effect of exercise training on appetite control. The benefit observed on Ad_pl_-to-fat mass ratio could likely reflect the effect of exercise training, either directly or through its effect on food intake.

Regarding Ad_mer_ distribution, the effect of exercising led to contrasting results in the literature. An absence of Ad_mer_ modulation upon training was observed by Ando et al. [61] after 12 weeks of combined resistance and aerobic exercise in Japanese participants. Other studies reported an increased HMW level after 12 weeks of aerobic exercise in obese adults with insulin resistance [62] and after 24 weeks of walking in obese middle-aged women [63]. Our results showed that the HMW proportion was significantly decreased in untrained HF mice but not in trained HF mice when compared with controls. However, the difference in the HMW proportion between the trained and untrained HF groups did not reach statistical significance. These discrepancies among studies could be related to the type and duration of exercise. Moreover, the different metabolic profiles observed in these studies could also have differently influenced the effect of training on Ad_mer_ distribution [61–63]. Regarding this point, Garekani et al. [59] and Chang et al. [64] suggested that the effect of exercise training on Ad_pl_ could depend on the obesity of the subject.

### An 8-week aerobic exercise training program increases AdipoR1 muscle expression level, an effect that is hampered by HF feeding

While the HF diet affected AdipoR2 levels, training increased the AdipoR1 expression level in muscle without modifying AdipoR2. This result was in agreement with the potential segregated functions of these receptors in muscle. Moreover, as only a slight and non-significant difference in body weight and food intake was observed between trained and control mice, it is conceivable to attribute AdipoR1 up-regulation to exercise training itself. This hypothesis is reinforced by the study of Goto et al. which observed a down-regulation of AdipoR1 mRNA in atrophied soleus muscle after a 2-week hindlimb suspension [65]. This alteration is no longer observed after a 2-week ambulation recovery protocol. Our data are thus in accordance with an AdipoR1 up-regulation induced by mechanical loading such as during exercise training.

Because of its role in metabolic homeostasis, the increase in AdipoR1 could contribute, at least partly, to the beneficial effect of exercise training. As it was reported that AdipoR1 expression is negatively regulated by insulin [14], it seems reasonable to hypothesize that AdipoR1 upregulation in SF trained mice may be consecutive to a lower insulin level, as mentioned by Huang et al. [66]. In transgenic animal models [64, 66] or obese subjects [62], previous studies have found an increase in muscular AdipoR1 mRNA [62, 66] and protein levels [55, 10] after physical activity. However, as Farias et al. found in skeletal muscle, we observed an opposite interaction of HF feeding and exercise training on AdipoR1 level regulation [67]. Molecular mechanisms involved in this interaction are still to determine, but FoxO and ATF3 signaling pathways should be considered for further investigations [14].

In HF-fed animals, if exercise training reduced glucose intolerance, the GTT response was not completely normalized to the level of the control animals. Simultaneously, we also observed that AdipoR1 upregulation by exercise is no longer present upon HF feeding. Although other mechanisms are most probably required, an association between both results may be considered. In addition, we also have to mention that in obese patients [57] and in HF-fed rats [68], skeletal muscle Ad resistance has been described. Although a downregulation of AdipoR has been suggested, the causes at the basis of this phenomenon are not completely known [69].

## Conclusion

In conclusion, the HF-fed mice recapitulated the key problems of MetS, despite compensatory mechanisms involving AdipoR2 muscle expression. Our study also showed that 8 weeks of exercise training could reduce adipose tissue dysfunction and induce an increased level of AdipoR1 in muscle. Upon HF feeding, a concomitant aerobic exercise training prevented numerous metabolic perturbations either directly or by modifying nutrition behavior. However, HF feeding during a training program hampered AdipoR1 upregulation in muscle. The consecutive attenuation of Ad signaling could partly limit the beneficial effects of exercise training on metabolism in the context of MetS. However, besides Ad pathway, additional processes are likely involved in the beneficial effects of exercise training on the metabolism of HF-fed animals and should be further examined. Finally, our study supports the growing evidence of divergent functions of AdipoR1 and AdipoR2 receptors. It also highlights that modulations of Ad_mer_ distribution and AdipoR expression should be taken into consideration for the diagnosis of MetS as well as for current therapeutic strategies that aim to increase circulating Ad levels.

## Additional files

Additional file 1:
**Figure S1.** Ad_mer_ distribution analysis. The method used is the same as that described in Fig. 5. (a and b) Admer/total Ad ratio obtained after densitometric analysis. Data are represented as boxplots. Panel a) diet effect, Panel b) training effect, * *p* < 0.05; ** *p* < 0.001, HF vs SF, Mann-Whitney rank sum test. **Figure S2.** Evaluation of the specificity and cross-reactivity of AdipoR antibodies by WB: peptide (pep) competition assay. **Table S1.** 70 % of the maximal running velocity of trained groups represented as mean ± SEM. **Table S2.** The imaging parameters for the 3D FISP sequence. **Table S3.** Primer sequences used in RTqPCR analysis (DOCX 513 kb)
Additional file 2: Raw data including weight, food intake, lean and fat mass, glucose tolerance, Ad mRNA expression in adipose tissue, Ad plasmatic concentration, Ad-mer distribution analysis, AdipoR1 protein expression and AdipoR2 protein expression. (XLSX 23 kb)

## Abbreviations

Ad: Adiponectin
AdipoR: Adiponectin receptor
Ad_mer_: Adiponectin multimers
Ad_pl_: Adiponectin plasmatic level
GTT: Glucose tolerance test
HF: High-fat high-sugar
HFC: Untrained mice submitted to a high-fat high-sugar diet
HFT: Trained mice submitted to a high-fat high-sugar diet
HMW: High molecular weight
LMW: Low molecular weight
MetS: Metabolic syndrome
MMW: Medium molecular weight
MRI: Magnetic resonance imaging
SF: Standard
SFC: Untrained mice submitted to a standard diet
SFT: Trained mice submitted to a standard diet
