# Supplementary material for: Interactions of exercise training and high-fat diet on adiponectin forms and muscle receptors in mice
Source: Nutr Metab (Lond). 2016 Nov 3;13:75. doi: 10.1186/s12986-016-0138-2 (PMC5094086; doi:10.1186/s12986-016-0138-2)
Supplement: Additional file 1: — Figure S1. Admer distribution analysis. The method used is the same as that described in Fig. 5. (a and b) Admer/total Ad ratio obtained after densitometric analysis. Data are represented as boxplots. Panel a) diet effect, Panel b) training effect, * p < 0.05; ** p < 0.001, HF vs SF, Mann-Whitney rank sum test. Figure S2. Evaluation of the specificity and cross-reactivity of AdipoR antibodies by WB: peptide (pep) competition assay. Table S1. 70 % of the maximal running velocity of trained groups represented as mean ± SEM. Table S2. The imaging parameters for the 3D FISP sequence. Table S3. Primer sequences used in RTqPCR analysis (DOCX 513 kb) [file 12986_2016_138_MOESM1_ESM.docx]

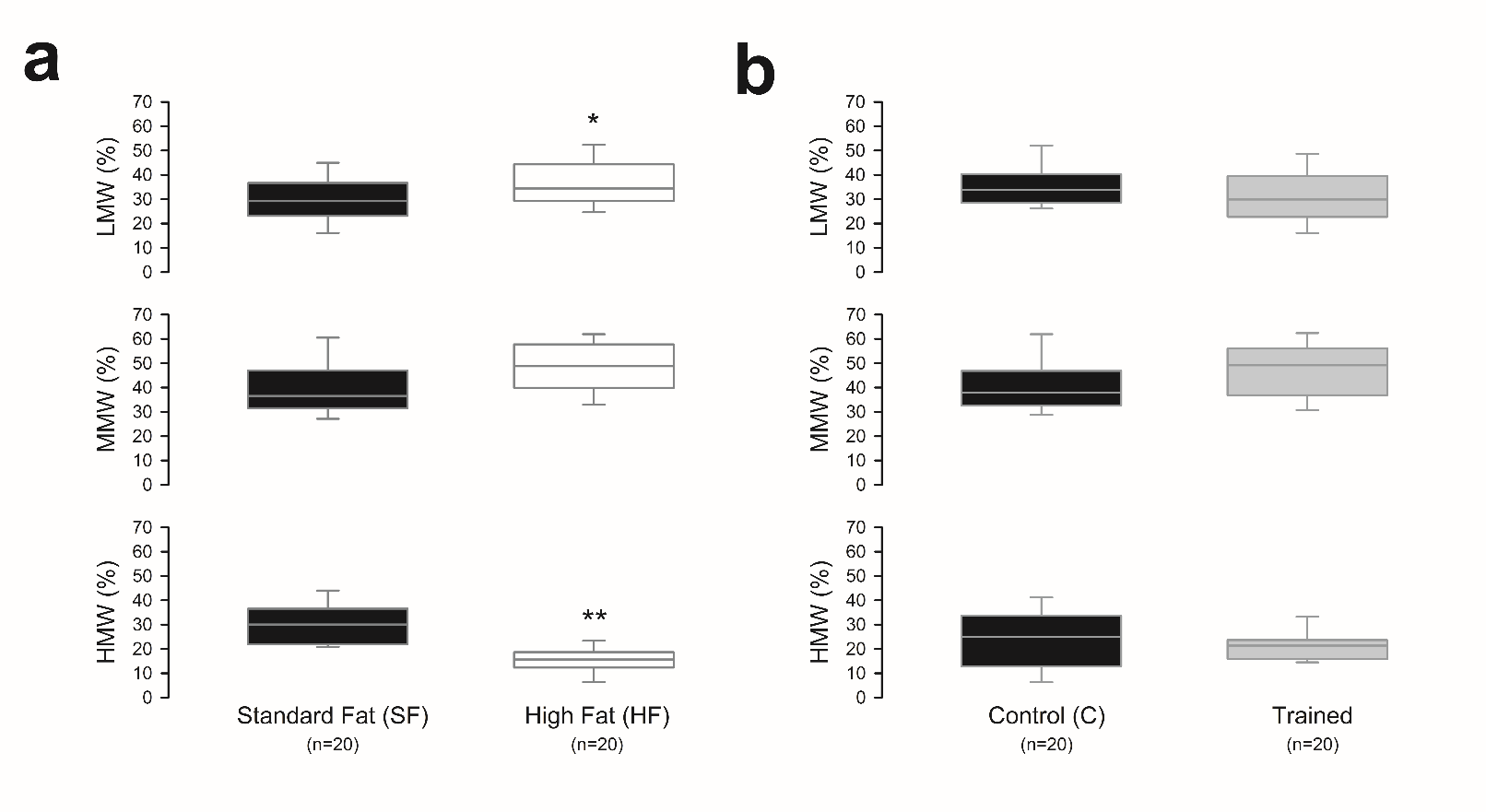


**Figure S1. Ad_mer_ distribution analysis .** The method used is the same as that was described in Figure 5. **(a and b)** **Admer/total Ad ratio** obtained after densitometric analysis. Data are represented as boxplots. Panel a) diet effect, Panel b) training effect, * p<0.05; ** p<0.001, HF vs SF, Mann-Whitney rank sum test.


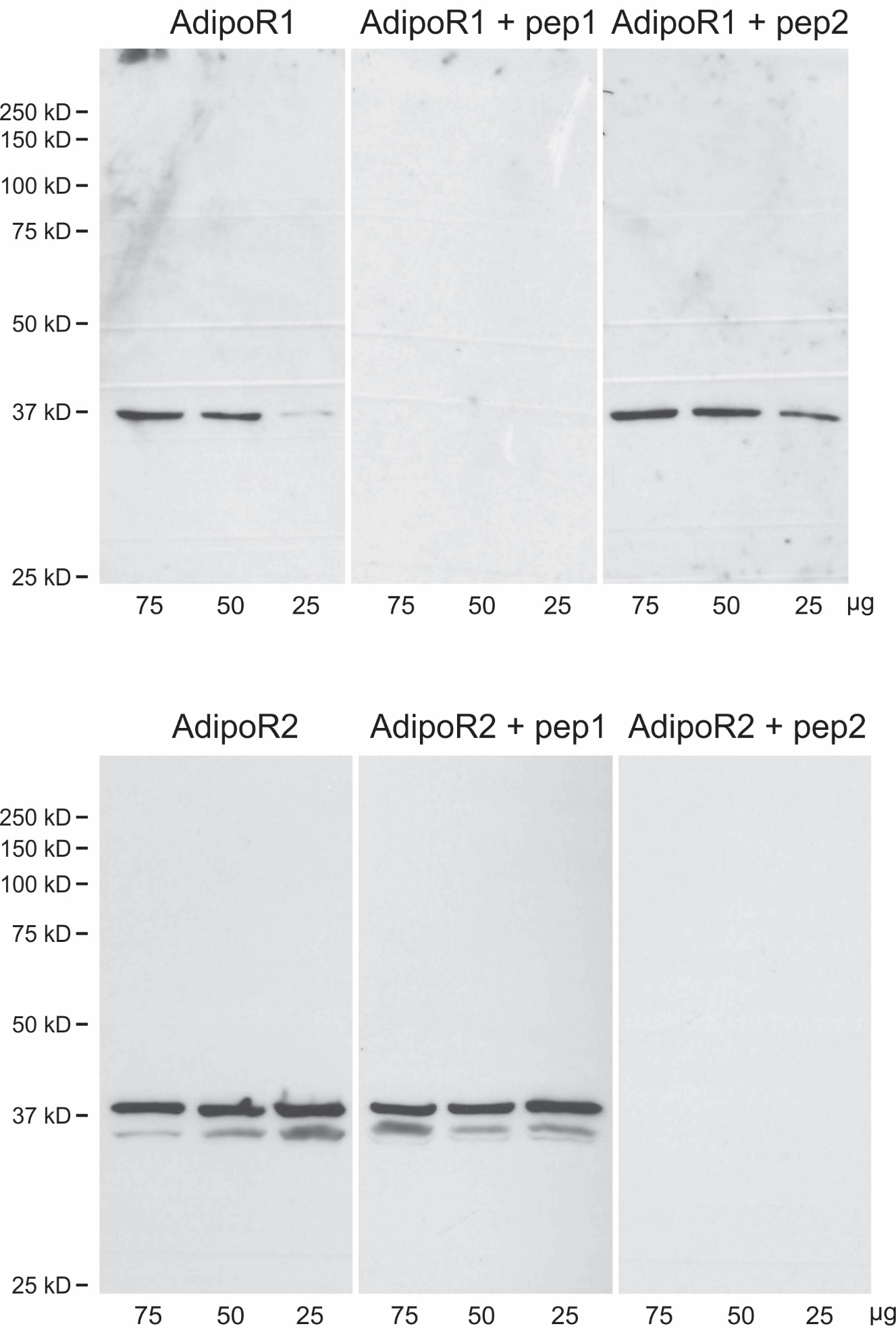


**Figure S2. Evaluation of the specificity and cross-reactivity of AdipoR antibodies by WB:** peptide (pep) competition assay.

**Table S1. 70% of the maximal running velocity of trained groups represented as mean ± SEM**

| SFT group | 16.7 ± 0.4 m/min |
| --- | --- |
| HFT group | 16.3 ± 0.3 m/min |

**Table S2. The imaging parameters for the 3D FISP sequence**

| repetition time | 3.4 ms |
| --- | --- |
| echo time | 1.7 ms |
| flip angle | 20° for muscle images and 45° for fat images |
| matrix size | 256x192x92 |
| field of view | 5x3x3 cm |
| spatial resolution | 195x156x326 µm |

**Table S3. Primer sequences used in RTqPCR analysis**

| Target genes | Primer sequences |
| --- | --- |
| Adiponectin | Forward: 5’ – GTTGCAAGCTCTCCTGTTCC – 3’  Reverse: 5’ – TCTCCAGGAGTGCCATCTCT – 3’ |
| GAPDH | Forward: 5’ – CTGCACCACCAACTGCTTAG - 3’  Reverse: 5’ – CTTCTGAGTGGCAGTGATGG - 3’ |
